# Dry Stamping Coral Powder: An Effective Method for Isolating Coral Symbiotic Actinobacteria

**DOI:** 10.3390/microorganisms11122951

**Published:** 2023-12-10

**Authors:** Amayaly Becerril-Espinosa, Carolina Mateos-Salmón, Asdrubal Burgos, Fabián A. Rodríguez-Zaragoza, Iván D. Meza-Canales, Eduardo Juarez-Carrillo, Eduardo Rios-Jara, Héctor Ocampo-Alvarez

**Affiliations:** 1 Consejo Nacional de Humanidades, Ciencias y Tecnologías (CONAHCYT), Mexico City 03940, Mexico; amayaly.becerril@academicos.udg.mx (A.B.-E.); asdrubal.burgos@academicos.udg.mx (A.B.); 2 Departamento de Ecología, Centro Universitario de Ciencias Biológicas y Agropecuarias, Universidad de Guadalajara, Zapopan 45200, Mexicofabian.rzaragoza@academicos.udg.mx (F.A.R.-Z.); ejuarez@cucba.udg.mx (E.J.-C.); eduardo.rios@academicos.udg.mx (E.R.-J.); 3 Departamento de Botánica y Zoología, Centro Universitario de Ciencias Biológicas y Agropecuarias, Universidad de Guadalajara, Zapopan 45200, Mexico; ivan.meza5024@academicos.udg.mx

**Keywords:** coral, marine actinobacteria, *Salinispora*, *Streptomyces*, symbiosis, coral actinobacteria

## Abstract

Actinobacteria are important sources of antibiotics and have been found repeatedly in coral core microbiomes, suggesting this bacterial group plays important functional roles tied to coral survival. However, to unravel coral–actinobacteria ecological interactions and discover new antibiotics, the complex challenges that arise when isolating symbiotic actinobacteria must be overcome. Moreover, by isolating unknown actinobacteria from corals, novel biotechnological applications may be discovered. In this study, we compared actinobacteria recovery from coral samples between two widely known methods for isolating actinobacteria: dry stamping and heat shock. We found that dry stamping was at least three times better than heat shock. The assembly of isolated strains by dry stamping was unique for each species and consistent across same-species samples, highlighting that dry stamping can be reliably used to characterize coral actinobacteria communities. By analyzing the genomes of the closest related type strains, we were able to identify several functions commonly found among symbiotic organisms, such as transport and quorum sensing. This study provides a detailed methodology for isolating coral actinobacteria for ecological and biotechnological purposes.

## 1. Introduction

Actinobacteria are prolific sources of antibiotics and bioactive natural products [1]. Recent metagenomic studies of coral bacterial communities have shown that actinobacteria are highly represented within several coral species, suggesting that actinobacteria form part of the coral core microbiome, making them essential to corals [2,3]. Furthermore, actinobacteria have been located in coral gastrodermal cells and even within other coral endosymbionts, including dinoflagellates of the genus *Symbiodinium* [4,5]. Given their importance, research exploring the multifaceted roles of actinobacteria is essential; however, methods for isolating symbiotic coral actinobacteria efficiently must first be developed and refined in order to capture the most abundant species and minorities with relevant ecological functions.

Actinobacteria exhibit diverse biochemical characteristics, suggesting these organisms play several functional roles in corals. For example, some actinobacteria exhibit strong enzymatic activity responsible for transforming recalcitrant molecules into ones that corals or their dinoflagellated symbionts can assimilate [6,7,8,9]. Other actinobacteria genera, such as *Frankiales* and *Streptomyces*, are well recognized for their ability to fix nitrogen and solubilize phosphate when interacting with plants [10,11,12]. In marine ecosystems, actinobacteria secrete secondary metabolites that inhibit pathogenic bacteria [13,14,15,16], suggesting actinobacteria play defensive roles within their hosts [14,15,17].

Further work is needed to gain deeper insights into the ecological functions of coral actinobacteria, opening the door to the discovery and development of novel biotechnological applications. For example, after learning that actinobacteria share a niche with a photosynthetic coral symbiont [4], we evaluated whether applying *Salinispora arenicola*, a symbiotic actinobacterium present in corals, would benefit the growth of terrestrial plants. We found that *S. arenicola* promoted the growth of tomato and wild tobacco plants while protecting them from salinity stress, highlighting its biotechnological potential for agricultural production [18,19]. In addition, the ability of actinobacteria to produce antibiotics is well known, with these organisms currently producing 45% of commercial antibiotics [20]. This ability can also be observed in corals, with actinobacteria maintaining pathogenic bacteria, such as *Vibrio coralliilyticus*, at safe levels by producing antibiotics [13]. Thus, bacterial consortiums containing actinobacteria could potentially be employed as probiotics to safeguard coral health in conservation strategies [21].

While coral symbiotic actinobacteria show great biotechnological potential, the major challenges associated with isolating actinobacteria effectively and efficiently must be overcome. To this end, traditional and artisanal methods must be refined and developed to selectively isolate coral symbiotic actinobacteria strains. The primary challenge associated with isolating actinobacteria is that actinobacteria cannot outcompete fast-growing bacteria. Therefore, actinobacteria isolation media must be nutrient-diluted to slow down bacterial growth. For example, the nutrients in actinobacteria isolation media (e.g., medium A1) are typically diluted to 1% of standard growth medium [22].

In addition to nutrient depletion, antibiotics, heat shock, desiccation, and chemical signaling have also been employed to isolate actinobacteria in growth media. For example, antibiotics, including gentamicin and streptomycin, and antifungal compounds, such as nystatin and cycloheximide, have been extensively employed, either alone or in combination, to develop media that favor the isolated growth of particular actinobacteria strains [23,24]. Given that actinobacteria can form resistance structures, such as spores, procedures that induce physical stress, such as desiccation or heat shock, have been commonly employed to eliminate fast-growing, non-sporulating bacteria while favoring the growth of sporulating actinobacteria [25]. In addition, to induce the germination of actinobacteria spores, chemical signaling employing L-valine, L-alanine, or casamino acids has also been commonly used to isolate and favor actinobacteria growth in media [26].

Corals rely on specific biochemical signals to interact with their bacterial communities. Thus, adding coral derivatives to growth media may improve the isolation of coral symbiotic actinobacteria. We evaluated two methods incorporating various commonly used isolation strategies to develop a standardized methodology for isolating actinobacteria from corals. To achieve this, we used samples from three coral species to evaluate the capacity of each method to isolate actinobacteria communities. We performed an in silico analysis on the isolated actinobacteria to highlight important molecular functions and gain insight into the roles the isolated actinobacteria might play in coral–actinobacteria symbiosis. Our results open the door to the generalized use of a dry stamping method to isolate functionally and biotechnologically important coral actinobacteria.

## 2. Materials and Methods

### 2.1. Biological Materials

**Figure 1:**
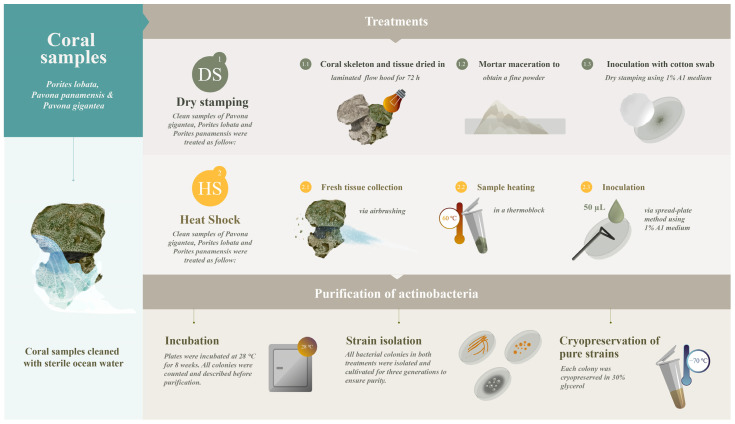
Experimental design for actinobacteria recovery by dry stamping (DS) and heat shock (HS). Coral tissues were diluted in water in the HS treatment.

Coral samples of healthy adult colonies of *Porites lobata* (PL), *Porites panamensis* (PP), and *Pavona gigantea* (PG) were collected from several tropical coral ecosystems in the central Mexican Pacific (19°5′55.21″, 104°23′24.47″) by scuba diving during the 2018 winter season. Coral samples were immediately stored at 4 °C and transported to the laboratory. Once in the laboratory, the seawater and mucus secretions on the surface of the coral samples were removed. The coral samples were then repeatedly washed with sterile seawater. The clean samples were used to evaluate the effectiveness of two methods, dry stamping and heat shock, in isolating symbiont actinobacteria (Figure 1).

### 2.2. Actinobacteria Isolation by Dry Stamping (DS)

The entire fraction of each mucus-free coral sample was dried in a laminated flow hood for 72 h. Subsequently, the dry coral samples were macerated in a mortar to obtain a fine powder. The A1 solid isolation medium, composed of 1.0 g of starch soluble, 0.1 g of casamino acids, 0.2 g of bactopeptone, 0.4 g of yeast extract, 18.0 g of Agar and 1.0 L of natural seawater, supplemented with 100 µg mL^−1^ of the antifungal cycloheximide and 5 µg mL^−1^ of the broad-spectrum antibiotic gentamicin previously sterilized via 0.2 µm filtration, was employed to prepare the Petri dishes [27]. To dry stamp the dried coral powder onto the A1 solid isolation medium in each Petri dish, a circular cotton swab was used as a paintbrush and impregnated with coral powder. The cotton swab was pressed repeatedly onto the solid isolation medium until the entire area of each Petri dish was covered in the dried powder. Then, the Petri dishes were incubated in darkness at 28 °C for eight weeks. The earliest actinobacteria colonies appeared four weeks after dry stamping. After eight weeks of incubation, the colonies were counted and described prior to isolation.

### 2.3. Actinobacteria Isolation by Heat Shock (HS)

The mucus-free coral samples were airbrushed (90 psi) with sterile seawater to obtain the coral tissue. Subsequently, the coral tissue was dispersed in seawater and heated (60 °C) for 10 min. Then, a 50 µL aliquot of heated coral tissue suspension was inoculated at the center of each Petri dish prepared with A1 solid isolation medium (see Section 2.2 for medium preparation). The coral suspension was distributed across the solid surface of the medium with a stainless steel Drigalski spatula. Subsequently, the Petri dishes were incubated at 28 °C for 8 weeks, and all colonies were counted and described before actinobacteria strain purification.

### 2.4. Actinobacteria Strain Purification

To ensure pure strains, after the initial 8-week incubation, all bacterial colonies in both treatments (i.e., DS and HS) were isolated and cultivated for three generations in A1 solid growth medium (18.0 g agar, 10.0 g starch, 2.0 g bactopeptone, 4.0 g yeast extract, 1 L natural seawater) free of gentamicin and cycloheximide to ensure their growth. Briefly, a sterile toothpick was used to carefully isolate and recover each bacterial colony, which was then inoculated in the new A1 medium. Actinobacteria strains were considered pure after the third clean growth on solid A1 media. Every single colony of each pure strain was inoculated into 50 mL of A1 liquid growth medium (10.0 g starch, 2.0 g bactopeptone, 4.0 g yeast extract, 1 L natural seawater) free of antibiotics and antifungals at 210 rpm, 28 °C for eight days. Pure strains were maintained by cryopreservation in 30% glycerol at −70 °C.

### 2.5. Strain Morphotypes

All purified actinobacteria strains were individually grown on solid A1 growth media at 28 °C until bacterial colonies were well developed (≈1 to 4 weeks). Thereafter, we evaluated the morphological and physiological characteristics of the colonies to classify morphotypes. To achieve this, we used colony color, shape, mycelium type, hyphae presence, diffusible pigment presence, and exudate production. The ability of each strain to grow at 0% and 3.5% NaCl was also evaluated to distinguish strict marine morphotypes from those that were halo-tolerant [27]. All strains were assayed using the KOH Gram-reaction method [28] to identify Gram-positive characteristics of the actinobacteria.

### 2.6. Phylogenetic Analysis of Actinobacteria

DNA was extracted from the isolated actinobacteria strains grown for eight days in liquid A1 medium at 28 °C with agitation (200 rpm). The commercial DNA isolation kit DNeasy ^®^ Blood and Tissue Kit Cat. N° 69,506 (Qiagen, Germantown, MD, USA) was used following the protocol of the manufacturer with modifications from Gontang et al. [22]. 16S ribosomal amplification was conducted with extracted DNA using the primers FC27 (5′-AGAGTTTGATCCTGGCTCAG-3′) and RC1492 (5′-TACGGCTACCTTGTTACGACTT-3′) and Dream Taq Green Master Mix (2X) (#K1081, Thermo Fisher Scientific, Waltham, MA, USA) in a total volume of 50 µL. The PCR reaction had an initial denaturation step of 95 °C for 15 min, followed by 32 amplification cycles (95 °C for 1 min, 61 °C for 1 min, and 72 °C for 1 min) and a final extension step of 72 °C for 7 min [22]. The commercial kit Wizard^®^ SV Gel and PCR Clean-Up System (Promega, Madison, WI, USA) was used to purify the PCR Products. The 16S ribosomal products were then sequenced at the DNA Synthesis and Sequencing Unit of the Institute of Biotechnology (National Autonomous University of Mexico, Cuernavaca, Mexico). 16S ribosomal sequences were assembled to a total of 1200–1400 bp and deposited in GenBank under the accession numbers OR711083–OR711103. The phylogenetic tree was constructed using the Neighbor-Joining method. Briefly, we employed 1300 base pairs, a bootstrap test (1000 replicates), the *p*-distance method, and the actinobacteria sequences of the closest type strains and *Bacillus acidicola* as outgroups. The sequences were multiple-aligned using ClustalX [29]. All analyses were conducted in MEGA7 [30].

### 2.7. Statistical Analysis

A two-way permutational analysis of variance (ANOVA) with crossed factors and fixed effects (model type I) was performed to evaluate variation in colony-forming units and the number of morphotypes among corals (PL, PP, and PG) and treatments (DS and HS). The permutational ANOVA was based on Euclidian distance following the criteria of Anderson et al. [31]. A principal coordinate analysis (PCoA) was constructed to explore variation in bacterial morphotype assemblages among corals (PL, PP, and PG) and treatments (DS and HS). Significant differences in actinobacteria assemblages were evaluated using a permutational multivariate analysis of variance (PERMANOVA) based on a Bray Curtis similarity matrix (model type 1). Results were considered statistically significant when *p* ≤ 0.05 using 10,000 residual permutations with a reduced model and sum of squares type III. Shadeplots and Venn diagrams are used to show our results [31,32].

### 2.8. Functional Analysis and Annotation

Functional analysis of the isolated strains was conducted using Piphillin [33] with the default settings. A list of the 16S sequences, colony number, and type per sample are provided in Supplementary Files S2 and S3. The keggGet function of the R package ‘*KEGGREST*’ [34] was used to annotate the list of functions provided by Piphillin. Descriptors brite3, brite4, brite5, and brite6 of the KEGG BRITE Database [35] were used because they are highly informative and available for all functions detected within the closest type strain genomes. The R script is available upon request.

## 3. Results

### 3.1. Recovery of Total Coral Actinobacteria Colonies in the DS and HS Treatments

**Figure 2:**
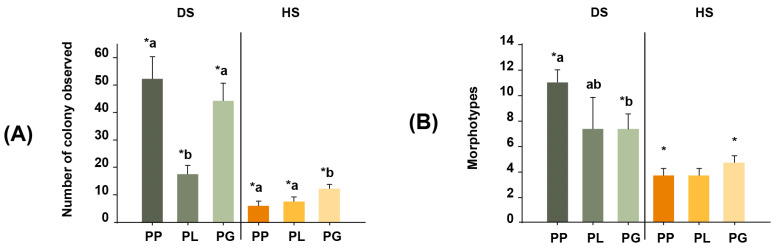
(**A**) Number of actinobacteria colonies observed for each of the three coral species: *Porites lobata* (PL), *Porites panamensis* (PP), and *Pavona gigantea* (PG). (**B**) Morphotypes of the actinobacteria colonies isolated from the three coral species. Treatments: DS (dry stamping) and HS (heat shock). Lowercase letters denote statistical differences among coral samples in each treatment. Symbols (*) denote statistical differences in the interactions between coral samples and treatment (*p* < 0.001).

The average colony counts of the DS samples were 52 colonies for PP, 17 for PL, and 44 for PG. In contrast, the average colony counts for the HS samples were much lower (6, 7, and 12 colonies for PP, PL, and PG, respectively). Indeed, the number of actinobacteria colonies recovered from PP samples via dry stamping was ~9 times greater than the number recovered via heat shock. Similarly, 2.3- and 3.6-fold more colonies were obtained for the PL and PG samples via dry stamping than via heat shock (Permutational ANOVA, Pseudo F = 190.83, *p* = 0.0001; Figure 2A).

### 3.2. Actinobacteria Morphotypes

**Figure 3:**
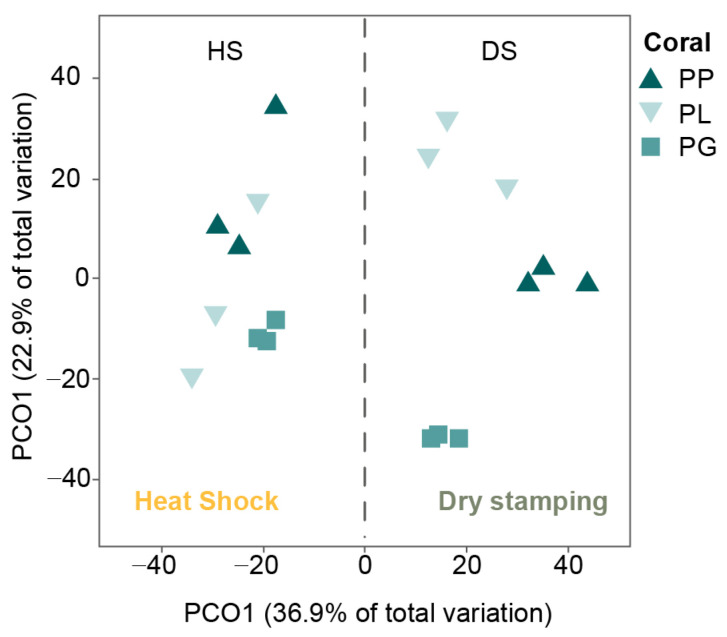
Principal coordinate analysis (PCoA) of the actinobacteria assemblages obtained from the three coral species: *Porites lobata* (PL), *Porites panamensis* (PP), and *Pavona gigantea* (PG). Treatments: DS (dry stamping) and HS (heat shock).

**Figure 4:**
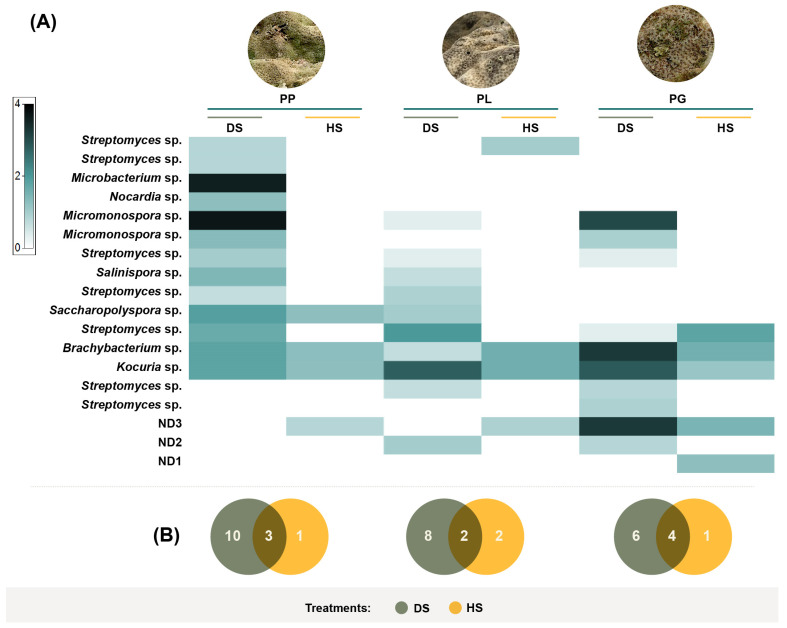
Shadeplot of the diversity and abundance of the taxonomically identified actinobacteria isolated from (**A**) *Porites panamensis* (PP), *Porites lobata* (PL), and *Pavona gigantea* (PG) in the (**B**) DS (dry stamping) and HS (heat shock) treatments.

The classification of actinobacteria colonies according to their morphological characteristics resulted in 18 morphotypes (Figure 2B), of which 16 were isolated via dry stamping, and only 6 were isolated via heat shock. When comparing the number of morphotypes recovered from the same coral species between the DS and HS treatments, dry stamping was superior to heat shock, with 6.8-, 3.8-, and 2.9-fold more colonies observed in PP, PL, and PG, respectively (PERMANOVA, Pseudo-F = 57.96, *p* = 0.0001). In addition, the diversity and abundance of actinobacteria morphotypes resulted in different assemblages between coral species (PERMANOVA, Pseudo-F = 11.139, *p* = 0.0001) and treatments (PERMANOVA, Pseudo-F = 29.843, *p* = 0.0002; Figure 3). Overall, dry stamping resulted in the recovery of more morphotypes than heat shock for all coral samples (Figure 4A,B).

### 3.3. Actinobacteria Phylogeny

**Figure 5:**
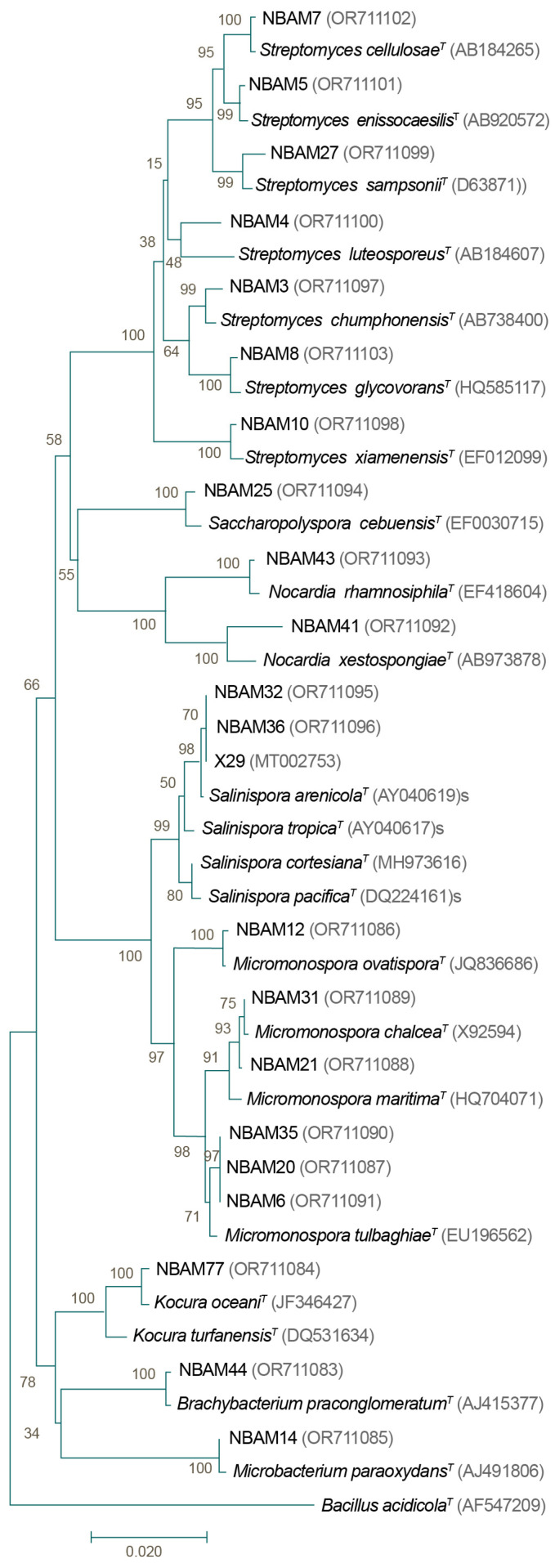
Phylogenetic tree of strains isolated from the three species of corals (*Porites lobata* (PL), *Porites panamensis* (PP), and *Pavona gigantea* (PG)) representing the main morphotypes isolated from the coral samples. Neighbor-Joining tree based on 16S rRNA gene sequences with 1300 base pairs employing a bootstrap test (1000 replicates), the *p*-distance method, and *Bacillus acidicola* as the outgroup. Accession numbers are shown in parentheses. The bar represents 2% sequence divergence. *^T^*: type strain. NBAM strains observed in this study.

BLAST searches of the 16S rRNA sequences of the isolated strains from every morphotype revealed a high similarity to one of six families: Dermabacteraceae (genus *Brachybacterium*), *Micromonosporaceae* (genera *Salinispora* and *Micromonospora*), *Mycobacterium* (genus *Microbacterium*), *Nocardiaceae* (genus *Nocardia*), *Pseudonocardiaceae* (genus *Saccharopolyspora*), and *Streptomycetaceae* (genus *Streptomyces*; Figure 5). The phylogenetic tree indicated that the strains shared 97.58–100% 16S rRNA sequence identity to the nearest actinobacteria type strain. The genus *Streptomyces* was highly representative since it was found in all coral samples, was the most diverse, and was represented by seven species. Strain NBAM4 reached a 97.58% sequence identity to a type strain of *S. luteosporeus* (accession number AB184607), and strain NBAM8 shared a 98.68% sequence identity with an *S. glycoforms* sequence type (accession number HQ585117). The other strains related to *Streptomyces* exhibited conserved sequence identity values higher than 99% to *S. cellulose*, *S. chumphonensis*, *S. enissocaesilis*, *S. Sampson*, and *S. xiamenensis*.

A *Salinispora* strain was found in PP and PL corals, which was related to *S. arenicola* (99.99% sequence identity to type strain AY040619). The strains of the genus *Micromonospora* were identified with 99.26–99.93% sequence identity to the species *M. chalcea*, *M. tulbaghiae*, and *M. ovatispora*. Two different species of *Nocardia* were found, with strain NBAM41 sharing 98.25% sequence identity with type strain *N. xestospongiae* (accession number AB973878), while strain NBAM43 was related to *N. rhamnosiphila* with 99.48% sequence identity. The remaining three morphotypes shared 99.55–99.71% sequence identity with *Saccharopolyspora cebuensis* (99.55% sequence identity with accession number EF030715), *Microbacterium paraoxydans* (99.92% sequence identity with accession number AJ491806), and *Brachybacterium paraconglomeratum* (99.71% sequence identity with accession number AJ415377).

### 3.4. Overrepresented Functions in Actinobacteria Genomes Recovered Using the HS and DS Treatments

**Figure 6:**
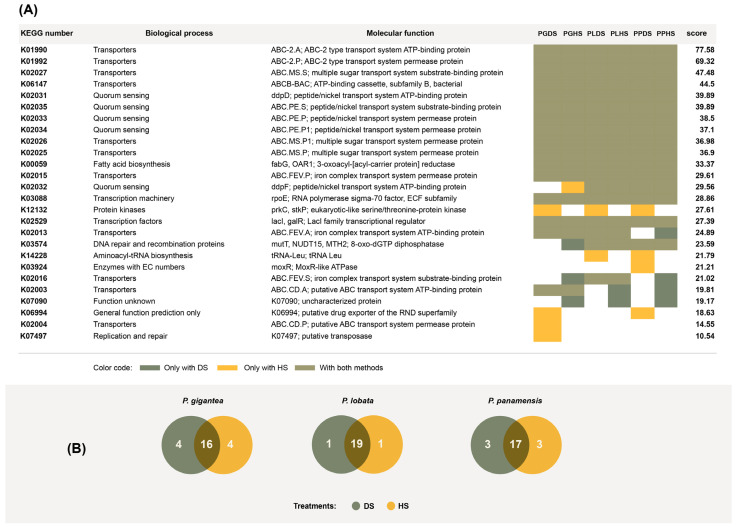
(**A**) Piphillin scores of the top 20 most overrepresented functions for all species and treatments. The scores are averages across all three species (*Porites lobata* (PL), *Porites panamensis* (PP), and *Pavona gigantea* (PG)) and treatments (DS (dry stamping) and HS (heat shock)). The list includes 26 elements, as there was no perfect overlap of the top 20 functions for all samples. (**B**) Overlap among the top 20 most overrepresented functions found in bacterial colonies isolated via dry stamping and heat shock in three different coral species.

We used Piphillin [33] to elucidate the functions of the actinobacteria present in the coral samples using the 16S ribosomal sequences isolated from PL, PG, and PS. Piphillin has previously been used for the functional annotation of prokaryotic genomes from a diverse range of biological samples or substrates [36,37,38]. Utilizing the list of 16S ribosomal sequences and the number of colonies per operational taxonomic unit, Piphillin returned a list of molecular functions contained in the genomes and their respective representations. Taking the top 20 most represented functions for each sample, we found notable overlap between the DS and HS treatments (Figure 6B). We also found notable overlap among the three coral species. Out of these functions, 10 were associated with transport, 5 were associated with quorum sensing, and 3 were related to transcription and translation (Figure 6A). We identified the genes K01990 and K01992 among transporter-related functions. These genes are orthologs of *yadG* and *yadH*, which are involved in antibiotic transport and have been reported to be upregulated in antibiotic-resistant *E. coli* [39]. K02025, K02026, and K02027 were also identified. These genes are orthologs of *ycjO*, *ycjP*, and *yjcN*, which are involved in ATP-dependent, ABC-type sugar transport [40].

## 4. Discussion

### 4.1. Recovery of Actinobacteria by Heat Shock and Dry Stamping

Isolating actinobacteria from hermatypic coral samples is considerably challenging and complex. In this study, we obtained 168 actinobacteria strains which we isolated from the samples of three coral species using two different techniques: dry stamping and heat shock. Dry stamping emulates the ideal conditions for actinobacteria spore formation and germination, and was more effective than heat shock in this study. By using dry stamping, we were able to stimulate dormant actinobacteria spores to germinate and grow while killing non-sporulating bacteria highly sensitive to dehydration, such as Gram-negative bacteria.

Heat shock is a frequent approach for isolating actinobacteria from terrestrial soil because it takes advantage of this bacterial group’s thermotolerance, which is superior to most Gram-negative soil bacteria [41]. Actinobacteria in terrestrial environments are subject to high thermal variability, having adapted to heat stress by forming thermoresistant spores. In contrast, marine environments experience far less temperature variability and exhibit more stable environmental conditions than terrestrial environments. Consequently, marine actinobacteria may not be as thermotolerant as their terrestrial counterparts. In this study, heat shock, which involved heating the coral samples to 60 °C to eliminate heat-susceptible bacteria, proved to be less effective than dry stamping in isolating coral actinobacteria. The disparity in thermotolerance between terrestrial and marine actinobacteria likely accounts for the lower efficiency of heat shock in recovering actinobacteria colonies from coral samples.

Isolating specific bacteria requires not only a medium that supports their growth but also the spatial separation of at least one colony from any possible contaminant. Therefore, the successful isolation of marine actinobacteria in this study was likely favored by the addition of antibiotics and antifungal agents, to reduce the probability of microbial contamination, and by the reduction of the nutrient concentration in the isolation medium, which slowed bacterial growth to facilitate the manual collection of pure colonies. Moreover, dry stamping allowed us to inoculate the actinobacterial cells in the samples, and also the full set of metabolites and essential chemical elements present in the corals. Therefore, if specific coral metabolites are necessary for bacterial growth, they were incorporated during inoculation by dry stamping the powder produced from the entire coral sample. Although comparisons of different pretreatments have not been conducted, modifications and chemical additions to media are commonly employed to isolate marine actinobacteria [42,43,44].

### 4.2. Dry Stamping Facilitates the Recovery of Coral Microbiome Actinobacteria

The effectiveness of dry stamping in isolating coral actinobacteria was evidenced by the recovery of different actinobacteria assemblages from the three different coral species (Figure 3). Metagenomics studies have reported a great diversity of coral bacteria [45] and species-specific communities [46]. Despite great advances in actinobacteria isolation from marine environments, isolating actinobacteria from corals remains challenging [47]. As many as 11 new actinobacteria species from nine families were recovered from corals up to 2022, and 12 of 13 new compounds discovered in the same period resulted biologically active, highlighting the great reservoir of potential specialized metabolites harboring the coral actinobacteria [48].

These are some of the few actinobacteria that have been isolated from corals: *Corynebacterium maris*, isolated from *Fungia granulosa* [6]; *Janibacter corallicola*, originally isolated from *Acropora gemmifera* [7]; and *Streptomyces corallincola* and *Kineosporia corallincola*, isolated from *Favites pentagona* [49].

The isolation of new actinobacteria species and strains expands our understanding of actinobacteria diversity, their functions within corals, and their biotechnological potential. For example, our laboratory group has successfully isolated, via dry stamping, a *Salinispora* strain that shows notable plant growth-promoting ability when applied to tomato and wild tobacco seedlings [18,19]. These findings highlight the necessity of standardized methodologies for isolating actinobacteria from corals in order to understand ecological activities in marine ecosystems and prospective biotechnological applications (in this case, agriculture production). Dry stamping has recently been used in echinoderms [50] and marine sediments [51] for actinobacteria separation for ecological and biotechnological study, demonstrating the adaptability of this technique.

### 4.3. Molecular Processes Necessary for Symbiosis Were Identified Based on the Functional Genome Annotation of the Actinobacteria Isolates

Although different actinobacteria assemblages were isolated from each coral species, the functional genomic analysis related to the closest type strain revealed conservation of overrepresented functions, suggesting that, regardless of symbiont composition, corals require actinobacteria that provide similar functions. Conversely, symbiotic actinobacteria may also require similar molecular machinery to successfully interact with coral cells.

The high number of transporters found among overrepresented functions is understandable because actinobacteria are endosymbionts and a high degree of host–symbiont interdependence relies on strong molecule exchange across membranes. We found two antibiotic transporters among the highest scoring functions (Figure 6). Antibiotic transport plays crucial roles in symbiotic actinobacteria, either to export antibiotics to the host or as a detoxification mechanism, requiring actinobacteria to tolerate self-produced antibiotics [52]. Quorum sensing was also found within the top twenty highest scoring functions. Quorum-sensing systems enable bacteria to withstand and interact with host defenses, making them crucial for establishing and sustaining symbiosis [53].

## 5. Conclusions

We have shown that dry stamping, which consists of macerating dried coral samples to produce a fine powder, in conjunction with growth medium modifications in the form of adding antimicrobials and limiting nutrients, is an effective method to isolate coral symbiont actinobacteria. Dry stamping is likely also useful when attempting to isolate actinobacteria from other marine organisms. Dry stamping allowed us to successfully isolate several strains of actinobacteria from *P. lobata*, *P. panamensis*, and *P. gigantea*. Successful isolation of different marine actinobacteria, such as we report in this study, will further research on actinobacteria, their diversity, symbiotic host interactions, and their biotechnological potential.

## Data Availability

Data are contained within the Supplementary Materials.

## Supplementary Materials

The following supporting information can be downloaded at: https://www.mdpi.com/article/10.3390/microorganisms11122951/s1. Table S1. All functions with their scores according to Piphillin analysis of the closest type strain of actinobacterial colonies recovered from *P. gigantea, P. lobata and P. panamensis* samples upon dry stamping (DS) or heat shock (HS); Table S2. Actinobacteria colonies observed; Sequences S3. Sequences of Actinobacteria
