# Supplementary material for: Dry Stamping Coral Powder: An Effective Method for Isolating Coral Symbiotic Actinobacteria
Source: Microorganisms. 2023 Dec 10;11(12):2951. doi: 10.3390/microorganisms11122951 (PMC10745815; doi:10.3390/microorganisms11122951)
Supplement: Supplementary file 1 [file microorganisms-11-02951-s001.zip › Sequences S3.pdf]

>otu1

ATGCAAGTCGAACGATGACGGTGGTGCTTGCACCaCcTGATTAGTGGCGAACGGgTGAGT

AACACGTGAGTAACCTGCCCtCCACTTCGGGATAACCTCGGGAAATCGTGGCTAATACCG

GATAcGAGCACTCATCGCATGGTGGGTGcTGGAAAGATTTATCGGTGGGGGATGGACTCG

CGGCCTATCAGTTTGTTGGTGAGGTGATGGCTCACCAAGACGATGACGGGTAGCCGGCCT

GAGAGGGCGACCGGCCACACTGGGACTGAGACACGGCCCAGACTCCTACGGGAGGCAGCA

GTGGGGAATATTGCACAATGGGCGAAAGCCTGATGCAGCGACGCCGCGTGGGGGATGACG

GCCTTCGGGTTGTAAACCCCTTTCAGTAGGGAAGAAGCGAGAGTGACGGTACCTGCAGAA

GAAGCGCCGGCTAACTACGTGCCAGCAGCCGCGGTAATACGTAGGGCGCAAGCGTTGTCC

GGAATTATTGGGCGTAAAGAGCTTGTAGGTGGCTTGTCGCGTCTGCCGTGAAAACCCGAG

GCTCAACCTCGGGCGTGCGGtGGGTACGGGCAGGCTAGAGTGTGGTAGGGGAGACTGGAA

CTCCTGGTGTAGCGGTGAAATGCGCAGATATCAGGAAGAACACCGATGGCGAAGGCAGGT

CTCTGGGCCATTACTGACACTGAGAAGCGAAAGCATGGGTAGCGAACAGGATTAGATACC

CTGGTAGTCCATGCCGTAAACGTTGGGCACTAGGTGTGGGGGACATTCCACGTTTTCCGC

GCCGTAGCTAACGCATTAAGTGCCCCGCCTGGGGAGTACGGCCGCAAGGCTAAAACTCAA

AGGAATTGACGGGGGCCCGCACAAGCGGCGGAGCATGCTGATTAATTCGATGCAACGCGA

AGAACCTTACCAAGGCTTGACATGCACTGGACGGCTGCAGAGATGTGGCTTTCTTTGGAC

TGGTGCACAGGTGGTGCATGGTTGTCGTCAGCTCGTGTCGTGAGATGTTGGGTTAAGTCC

CGCAACGAGCGCAACCCTTGTTCTATGTTGCCAGCACGTGATGGTGGGGACTCATAGGAG

ACTGCCGGGGTCAACTCGGAGGAAGGTGGGGACGACGTCAAATCATCATGCCCCTTATGT

CTTGGGCTTCAAGCATGCTACAATGGTCGGTACAATGGGTTGCGAAACTGTGAGGTGGAG

CGAATCCCAAAAAGCCGGCCTCAGTTCGGATTGGGGTCTGCAACTCGACCCCATGAAGTC

GGAGTCGCTAGTAATCGCAGATCAGCAACGCTGCGGTGAATACGTTCCCGGGCCTTGTAC

ACA

>otu2

TGCAAGTCGAACGCTGAAGCATCTTCGGGTGTGGATGAGTGGCGAACGGGTGAgTAACAC

GTGGGTAATCTGCCCTGCACTCTGGGATAAGCCCTGGAAACGGGGTCTAATACCGGATAG

GACATTCTGCCGCATGGTGGGGTGTGGAAAGTTCCGGCGGTGCAGGATGAGCCCGCGGCC

TATCAGCTTGTTGGTGGGGTGATGGCCTACCAAGGCGACGACGGGTAGCCGGCCTGAGAG

GGTGACCGGCCACACTGGGACTGAGACACGGCCCAGACTCCTACGGGAGGCAGCAGTGGG

GAATCTTGCGCAATGGGCGAAAGCCTGACGCAGCAACGCCGCGTGGGGGATGACGGCCTT

CGGGTTGTAAACCTCTTTCGACAGGGACGAAGCTTTCGGGTGACGGTACCTGTAGAAGAA

GCACCGGCTAACTACGTGCCAGCAGCCGCGGTAATACGTAGGGTGCGAGCGTTGTCCGGA

TTTATTGGGCGTAAAGAGCTCGTAGGCGGTTTGTCGCGTCGGCCGTGAAAACCTGCAGCT

TAACTGTGGGCGTGCGGTCGATACGGGCAGACTTGAGTTCGGCAGGGGAGACTGGAATTC

CTGGTGTAGCGGTGAAATGCGCAGATATCAGGAGGAACACCGGTGGCGAAGGCGGGTCTC

TGGGCCGATACTGACGCTGAGGAGCGAAAGCGTGGGGAGCGAACAGGATTAGATACCCTG

GTAGTCCACGCCGTAAACGTTGGGCGCTAGGTGTGGGGACCGGTTCCACGGTTTCTGTGC

CGTAGCTAACGCATTAAGCGCCCCGCCTGGGGAGTACGGCCGCAAGGCTAAAACTCAAAG

GAATTGACGGGGGCCCGCACAAGCGGCGGAGCATGTGGATTAATTCGATGCAACGCGAAG

AACCTTACCTGGGTTTGACATGCACCAGATTGCCCCTGAGAGGGGGTTTCCCTTGTGGTT

GGTGTACAGGTGGTGCATGGCTGTCGTCAGCTCGTGTCGTGAGATGTTGGGTTAAGTCCC

GCAACGAGCGCAACCCTTATCCTGTGTTGCCAGCACGTAATGGTGGGGACTCGCGGGAGA

CTGCCGGGGTCAACTCGGAGGAAGGTGGGGATGACGTCAAGTCATCATGCCCCTTATGCC

CAGGGCTTCACACATGCTACAATGGCTGGTACAGAGGGTGGCGATACCGTGAGGTGGAGC

GAATCCCTTAAAGCCGGTCTCAGTTCGGATCGGGGTCTGCAACTCGACCCCGTGAAGTCG

GAGTCGCTAGTAATCGCAGATCAGCAGTGCTGCGGTGAATACGTTCCCGGGCCTTGTACA

CAC

>otu3

AAGTCGAGCGGAAAGGCCCTTCGGGGTACTCGAGCGGCGAACGGGTGAGTAACACGTGAG

CAACCTGCCCTAGGCTTTGGGATAACCCTCGGAAACGGGGGCTAATACCGGATATCACAT

TCTTCCGCATGGTTGGGTGTTGAAAGTTTTTCGGCCTGGGATGGGCTCGCGGCCTATCAG

CTTGTTGGTGGGGTGATGGCCTACCAAGGCGACGACGGGTAGCCGGCCTGAGAGGGCGAC

CGGCCACACTGGGACTGAGACACGGCCCAGACTCCTACGGGAGGCAGCAGTGGGGAATAT

TGCACAATGGGCGGAAGCCTGATGCAGCGACGCCGCGTGAGGGATGACGGCCTTCGGGTT

GTAAACCTCTTTCAGCAGGGACGAAGCGTAAGTGACGGTACCTGCAGAAGAAGCGCCGGC

CAACTACGTGCCAGCAGCCGCGGTAAGACGTAGGGCGCGAGCGTTGTCCGGATTTATTGG

GCGTAAAGAGCTCGTAGGCGGCTTGTCGCGTCGACTGTGAAAACCCGCAGCTCAACTGCG

GGCCTGCAGCCGATACGGGCAGGCTAGAGTTCGGTAGGGGAGACTGGAATTCCTGGTGTA

GCGGTGAAATGCGCAGATATCAGGAGGAACACCGGTGGCGAAGGCGGGTCTCTGGGCCGA

TACTGACGCTGAGGAGCGAAAGCGTGGGGAGCGAACAGGATTAGATACCCTGGTAGTCCA

CGCTGTAAACGTTGGGCGCTAGGTGTGGGGGGCCTCTCCGGTTCTCTGTGCCGCAGCTAA

CGCATTAAGCGCCCCGCCTGGGGAGTACGGCCGCAAGGCTAAAACTCAAAGGAATTGACG

GGGGCCCGCACAAGCGGCGGAGCATGCGGATTAATTCGATGCAACGCGAAGAACCTTACC

TGGGTTTGACATGGGCGGAAATCCGGCAGAGATGTCGGGTCCTTCGGGGCCGTTCACAGG

TGGTGCATGGCTGTCGTCAGCTCGTGTCGTGAGATGTTGGGTTAAGTCCCGCAACGAGCG

CAACCCTTATTCGATGTTGCCAGCGCGTTATGGCGGGGACTCATCGAAGACTGCCGGGGT

CAACTCGGAGGAAGGTGGGGATGACGTCAAGTCATCATGCCCCTTATGTCCAGGGCTTCA

CGCATGCTACAATGGCCGGTACAATGGGCTGCGATACCGTGAGGTGGAGCGAATCCCAAA

AAGCCGGTCTCAGTTCGGATCGGGGTCTGCAACTCGACCCCGTGAAGTCGGAGTCGCTAG

TAATCGCAGATCAGCAACGCTGCGGTGAATACGTTCCCGGGCCTTGTACACACCGCCCGT

CAC

>otu4

AAGTCGAACGATGAACCGGTTTCGGCCGGGGATTAGTGGCGAACGGGTGAGTAACACGTG

GGCAATCTGCCCTGCACTCTGGGATAAGCCCGGGAAACTGGGTCTAATACCGGATACGAC

ACATGAGGGCATCCTCGTGTGTGGAAAGTTCCGGCGGTGCAGGATGAGCCCGCGGCCTAT

CAGCTTGTTGGTGGGGTAGTGGCCTACCAAGGCGACGACGGGTAGCCGGCCTGAGAGGGT

GACCGGCCACACTGGGACTGAGACACGGCCCAGACTCCTACGGGAGGCAGCAGTGGGGAA

TATTGCACAATGGGCGAAAGCCTGATGCAGCGACGCCGCGTGAGGGATGACGGCCTTCGG

GTTGTAAACCTCTTTCAGCAGGGAAGAAGCGAAAGTGACGGTACCTGCAGAAGAAGCACC

GGCTAACTACGTGCCAGCAGCCGCGGTAATACGTAGGGTGCAAGCGTTGTCCGGAATTAT

TGGGCGTAAAGAGCTCGTAGGCGGCCTGTCGCGTCGATTGTGAAAGCCCAGGGCTTAACC

CTGGGTCTGCAGTCGATACGGGCAGGCTAGAGTTCGGTAGGGGAGATCGGAATTCCTGGT

GTAGCGGTGAAATGCGCAGATATCAGGAGGAACACCGGTGGCGAAGGCGGATCTCTGGGC

CGATACTGACGCTGAGGAGCGAAAGCGTGGGGAGCGAACAGGATTAGATACCCTGGTAGT

CCACGCCGTAAACGGTGGGCACTAGGTGTGGGCAACATTCCACGTTGTCCGTGCCGCAGC

TAACGCATTAAGTGCCCCGCCTGGGGAGTACGGCCGCAAGGCTAAAACTCAAAGGAATTG

ACGGGGGCCCGCACAAGCGGCGGAGCATGTGGCTTAATTCGACGCAACGCGAAGAACCTT

ACCAAGGCTTGACATACACCGGAAACGGCCAGAGATGGTCGCCCCCTTGTGGTCGGTGTA

CAGGTGGTGCATGGCTGTCGTCAGCTCGTGTCGTGAGATGTTGGGTTAAGTCCCGCAACG

AGCGCAACCCTTATCCTGTGTTGCCAGCGGATCCCTTCGGGGGTGCCGGGGACTCACGGG

AGACTGCCGGGGTCAACTCGGAGGAAGGTGGGGACGACGTCAAGTCATCATGCCCCTTAT

GTCTTGGGCTGCACACGTGCTACAATGGCCGGTACAATGAGCTGCGATACCGCGAGGTGG

AGCGAATCTCAAAAAGCCGGTCTCAGTTCGGATTGGGGTCTGCAACTCGACCCCATGAAG

TCGGAGTCGCTAGTAATCGCAGATCAGCATTGCTGCGgtGAATACGTTCCCGGGCCTTGT

ACA

>otu5

CATGCAAGTCGAACGATGAAGCCCTTTCGGGGGTGGATTAGTGGCGAACGGGTGAGTAAC

ACGTGGGCAATCTGCCCTGCACTTCGGGACAAGCCCTGGAAACGGGGTCTAATACCGGAT

ACGACTCGGGGAGGCATCTCCTCCGGGTGGAAAGCTTCGGCGGTGCAGGATGAGCCCGCG

GCCTATCAGCTTGTTGGTGGGGTGATGGCCTACCAAGGCGACGACGGGTAGCCGGCCTGA

GAGGGCGACCGGCCACACTGGGACTGAGACACGGCCCAGACTCCTACGGGAGGCAGCAGT

GGGGAATATTGCACAATGGGCGGAAGCCTGATGCAGCGACGCCGCGTGAGGGATGACGGC

CTTCGGGTTGTAAACCTCTTTCAGCAGGGAAGAAGCTTTTGTGACGGTACCTGCAGAAGA

AGCACCGGCTAACTACGTGCCAGCAGCCGCGGTAATACGTAGGGTGCGAGCGTTGTCCGG

AATTATTGGGCGTAAAGAGCTCGTAGGCGGCCTGTCGCGTCGGATGTGAAAGCCCGGGGC

TTAACCCTGGGTCTGCATTCGATACGGGCAGGCTGGAGTTCGGTAGGGGAGATCGGAATT

CCTGGTGTAGCGGTGAAATGCGCAGATATCAGGAGGAACACCGGTGGCGAAGGCGGATCT

CTGGGCCGATACTGACGCTGAGGAGCGAAAGCATGGGGAGCGAACAGGATTAGATACCCT

GGTAGTCCATGCCGTAAACGTTGGGCACTAGGTGTGGGCGACATTCCACGTTGTCCGTGC

CGCAGCTAACGCATTAAGTGCCCCGCCTGGGGAGTACGGCCGCAAGGCTAAAACTCAAAG

GAATTGACGGGGGCCCGCACAAGCGGCGGAGCATGTGGCTTAATTCGACGCAACGCGAAG

AACCTTACCAAGGCTTGACATACATCAGAAAGCTGTGGAGACACAGCCTCCCTTGTGGCT

GGTGTACAGGTGGTGCATGGCTGTCGTCAGCTCGTGTCGTGAGATGTTGGGTTAAGTCCC

GCAACGAGCGCAACCCTTGTCTCGTGTTGCCAGCAGGCCCTTGTGGTGCTGGGGACTCAC

GGGAGACTGCCGGGGTCAACTCGGAGGAAGGTGGGGACGACGTCAAGTCATCATGCCCCT

TATGTCTTGGGCTGCACACGTGCTACAATGGCCGGTACAATGAGTGGCGATGCCGTGAGG

TGGAGCGAATCTCAAAAAGCCGGTCTCAGTTCGGATTGGGGTCTGCAACTCGACCCCATG

AAGTCGGAGTCGCTAGTAATCGCAGATCAGCATTGCTGCGGTGAATACGTTCCCGGGCCT

TGT

>otu6

CATGCAGTCGAACGATGAAGCCGCTTCGGTGGTGGATTAGTGGCGAACGGGTGAGTAACA

CGTGGGCAATCTGCCCTGCACTCTGGGACAAGCCCTGGAAACGGGGTCTAATACCGGATA

CGACaCAGGAAGGCATCTTCTCTGTGTGGAAAGCTCCGGCGGTGCAGGATGAGCCCGCGG

CCTATCAGCTTGTTGGTGGGGTGATGGCCTACCAAGGCGACGACGGGTAGCCGGCCTGAG

AGGGCGACCGGCCACACTGGGACTGAGACACGGCCCAGACTCCTACGGGAGGCAGCAGTG

GGGAATATTGCACAATGGGCGCAAGCCTGATGCAGCGACGCCGCGTGAGGGATGACGGCC

TTCGGGTTGTAAACCTCTTTCAGCAGGGAAGAAGCGcAAGTGACGGTACCTGCAGAAGAA

GCACCGGCTAACTACGTGCCAGCAGCCGCGGTAATACGTAGGGTGCGAGCGTTGTCCGGA

ATTATTGGGCGTAAAGAGCTCGTAGGCGGCTTGTCACGTCGGATGTGAAAGCCCGGGGCT

TAACCCCGGGTCTGCATTCGATACGGGCAGGCTAGAGTTCGGTAGGGGAGATCGGAATTC

CTGGTGTAGCGGTGAAATGCGCAGATATCAGGAGGAACACCGGTGGCGAAGGCGGATCTC

TGGGCCGATACTGACGCTGAGGAGCGAAAGCGTGGGGAGCGAACAGGATTAGATACCCTG

GTAGTCCACGCCGTAAACGTTGGGAACTAGGTGTGGGCGACATTCCACGTCGTCCGTGCC

GCAGCTAACGCATTAAGTTCCCCGCCTGGGGAGTACGGCCGCAAGGCTAAAACTCAAAGG

AATTGACGGGGGCCCGCACAAGCGGCGGAGCATGTGGCTTAATTCGACGCAACGCGAAGA

ACCTTACCAAGGCTTGACATACATCGGAAAGCCGTAGAGATACGGCCCCCCTTGTGGTCG

GTGTACAGGTGGTGCATGGCTGTCGTCAGCTCGTGTCGTGAGATGTTGGGTTAAGTCCCG

CAACGAGCGCAACCCTTGTTCTGTGTTGCCAGCATGCCTTTCGGGGTGATGGGGACTCAC

AGGAGACTGCCGGGGTCAACTCGGAGGAAGGTGGGGACGACGTCAAGTCATCATGCCCCT

TATGTCTTGGGCTGCACACGTGCTACAATGGCCGGTACAATGAGTTGCGATGCCGTGAGG

TGGAGCGAATCTCAAAAAGCCGGTCTCAGTTCGGATTGGGGTCTGCAACTCGACCCCATG

AAGTCGGAGTCGCTAGTAATCGCAGATCAGCATTGCTGCGGTGAATACGTTCCCGGGCCT

TGT

>otu7

ATGCAAGTCGAACGATGAACCACTTCGGTGGGGATTAGTGGCGAACGGGTGAGTAACACG

TGGGCAATCTGCCCTGCACTCTGGGACAAGCCCTGGAAACGGGGTCTAATACCGGATACT

GATCCTCGCAGGCATCTGCGAGGaTCGAAAGCTCCGGCGGTGCAGGATGAGCCCGCGGCC

TATCAGCTAGTTGGTGAGGTAACGGCTCACCAAGGCGACGACGGGTAGCCGGCCTGAGAG

GGCGACCGGCCACACTGGGACTGAGACACGGCCCAGACTCCTACGGGAGGCAGCAGTGGG

GAATATTGCACAATGGGCGAAAGCCTGATGCAGCGACGCCGCGTGAGGGATGACGGCCTT

CGGGTTGTAAACCTCTTTCAGCAGGGAAGAAGCGAAAGTGACGGTACCTGCAGAAGAAGC

GCCGGCTAACTACGTGCCAGCAGCCGCGGTAATACGTAGGGCGCAAGCGTTGTCCGGAAT

TATTGGGCGTAAAGAGCTCGTAGGCGGCTTGTCACGTCGGTTGTGAAAGCCCGGGGCTTA

ACCCCGGGTCTGCAGTCGATACGGGCAGGCTAGAGTTCGGTAGGGGAGATCGGAATTCCT

GGTGTAGCGGTGAAATGCGCAGATATCAGGAGGAACACCGGTGGCGAAGGCGGATCTCTG

GGCCGATACTGACGCTGAGGAGCGAAAGCGTGGGGAGCGAACAGGATTAGATACCCTGGT

AGTCCACGCCGTAAACGGTGGGCACTAGGTGTGGGCAACATTCCACGTTGTCCGTGCCGC

AGCTAACGCATTAAGTGCCCCGCCTGGGGAGTACGGCCGCAAGGCTAAAACTCAAAGGAA

TTGACGGGGGCCCGCACAAGCGGCGGAGCATGTGGCTTAATTCGACGCAACGCGAAGAAC

CTTACCAAGGCTTGACATACACCGGAAAACCCTGGAGACAGGGTCCCCCTTGTGGTCGGT

GTACAGGTGGTGCATGGCTGTCGTCAGCTCGTGTCGTGAGATGTTGGGTTAAGTCCCGCA

ACGAGCGCAACCCTTGTCCCGTGTTGCCAGCAGGCCCTTGTGGTGCTGGGGACTCACGGG

AGACCGCCGGGGTCAACTCGGAGGAAGGTGGGGACGACGTCAAGTCATCATGCCCCTTAT

GTCTTGGGCTGCACACGTGCTACAATGGCCGGTACAATGAGCTGCGATACCGCGAGGTGG

AGCGAATCTCAAAAAGCCGGTCTCAGTTCGGATTGGGGTCTGCAACTCGACCCCATGAAG

TCGGAGTCGCTAGTAATCGCAGATCAGCATTGCTGCGGTGAATACGTTCCCGGGCCTTGT

ACA

>otu8

GAACGATGAACCGCTTTCGGGCGGGGATTAGTGGCGAACGGGTGAGTAACACGTGGGCAA

TCTGCCCTGCACTCTGGGACAAGCCCTGGAAACGGGGTCTAATACCGGATATGACTGTCT

GCCGCATGGTGGATGGTGTAAAGCTCCGGCGGTGCAGGATGAGCCCGCGGCCTATCAGCT

TGTTGGTGAGGTAGTGGCTCACCAAGGCGACGACGGGTAGCCGGCCTGAGAGGGCGACCG

GCCACACTGGGACTGAGACACGGCCCAGACTCCTACGGGAGGCAGCAGTGGGGAATATTG

CACAATGGGCGAAAGCCTGATGCAGCGACGCCGCGTGAGGGATGACGGCCTTCGGGTTGT

AAACCTCTTTCAGCAGGGAAGAAGCGAAAGTGACGGTACCTGCAGAAGAAGCGCCGGCTA

ACTACGTGCCAGCAGCCGCGGTAATACGTAGGGCGCAAGCGTTGTCCGGAATTATTGGGC

GTAAAGAGCTCGTAGGCGGCTTGTCACGTCGGTTGTGAAAGCCCGGGGCTTAACCCCGGG

TCTGCAGTCGATACGGGCAGGCTAGAGTTCGGTAGGGGAGATCGGAATTCCTGGTGTAGC

GGTGAAATGCGCAGATATCAGGAGGAACACCGGTGGCGAAGGCGGATCTCTGGGCCGATA

CTGACGCTGAGGAGCGAAAGCGTGGGGAGCGAACAGGATTAGATACCCTGGTAGTCCACG

CCGTAAACGGTGGGCACTAGGTGTGGGCAACATTCCACGTTGTCCGTGCCGCAGCTAACG

CATTAAGTGCCCCGCCTGGGGAGTACGGCCGCAAGGCTAAAACTCAAAGGAATTGACGGG

GGCCCGCACAAGCGGCGGAGCATGTGGCTTAATTCGACGCAACGCGAAGAACCTTACCAA

GGCTTGACATACACCGGAAACGTCTGGAGACAGGCGCCCCCTTGTGGTCGGTGTACAGGT

GGTGCATGGCTGTCGTCAGCTCGTGTCGTGAGATGTTGGGTTAAGTCCCGCAACGAGCGC

AACCCTTGTCCCGTGTTGCCAGCAGGCCCTTTGGTGCTGGGGACTCACGGGAGACCGCCG

GGGTCAACTCGGAGGAAGGTGGGGACGACGTCAAGTCATCATGCCCCTTATGTCTTGGGC

TGCACACGTGCTACAATGGCCGGTACAATGAGCTGCGATACCGCGAGGTGGAGCGAATCT

CAAAAAGCCGGTCTCAGTTCGGATTGGGGTCTCCACTCGACCCCATGAAGTCGGAGTCGC

TAGTAATCGCAGATCAGCATTGCTGCGGTGAATACGTTCCCGGGCCTTTTACACTCCGCC

CGT

>otu9

AGTCGAGCGGAAAGGCCCTTCGGGGTACTCGAGCGGCGAACGGGTGAGTaacACGtGAGT

AACCTGCCCCAGGCTTTGGGATAACCCCGGGAAACCGGGGCTAATACCGGATATGACCAT

CTGTCGCATGGTGGGTGGTGGAAAGATTTTTTGGCTTGGGATGGGCTCGCGGCCTATCAG

CTTGTTGGTGGGGTGATGGCCTACCAAGGCGGCGACGGGTAGCCGGCCTGAGAGGGCGAC

CGGCCACACTGGGACTGAGACACGGCCCAGACTCCTACGGGAGGCAGCAGTGGGGAATCT

TGCACAATGGGCGGAAGCCTGATGCAGCGACGCCGCGTGAGGGATGACGGCCTTCGGGTT

GTAAACCTCTTTCAGCAGGGACGAAGCGTTTGTGACGGTACCTGCAGAAGAAGCGCCGGC

CAACTACGTGCCAGCAGCCGCGGTAAGACGTAGGGCGCAAGCGTTGTCCGGATTTATTGG

GCGTAAAGAGCTCGTAGGCGGCTTGTCGCGTCGACTGTGAAAACCCGTGGCTCAACTGCG

GGCTTGCAGGCGATACGGGCAGGCTAGAGTTCGGTAGGGGAGACTGGAATTCCTGGTGTA

GCGGTGAAATGCGCAGATATCAGGAGGAACACCGGTGGCGAAGGCGGGTCTCTGGGCCGA

TACTGACGCTGAGGAGCGAAAGCGTGGGGAGCGAACAGGATTAGATACCCTGGTAGTCCA

CGCTGTAAACGTTGGGCGCTAGGTGTGGGGGGCCTCTCCGGTTCTCTGTGCCGCAGCTAA

CGCATTAAGCGCCCCGCCTGGGGAGTACGGCCGCAAGGCTAAAACTCAAAGGAATTGACG

GGGGCCCGCACAAGCGGCGGAGCATGCGGATTAATTCGATGCAACGCGAAGAACCTTACC

TGGGTTTGACATCGCCGGAAATCCTTCAGAGATGGGGGGTCCtTCGGGGCCGGTGACAGG

TGGTGCATGGCTGTCGTCAGCTCGTGTCGTGAGATGTTGGGTTAAGTCCCGCAACGAGCG

CAACCCTTGTTCGATGTTGCCAGCGCGTTATGGCGGGGACTCATCGAAGACTGCCGGGGT

CAACTCGGAGGAAGGTGGGGATGACGTCAAGTCATCATGCCCCTTATGTCCAGGGCTTCA

CGCATGCTACAATGGCCGGTACAGTGGGCTGCGATACCGTGAGGTGGAGCGAATCCCAAA

AAGCCGGTCTCAGTTCGGATCGGGGTCTGCAACTCGACCCCGTGAAGTCGGAGTCGCTAG

TAATCGCAGATCAGCAACGCTGCGGTGAATACGTTCCCGGGCCTTGTaCACACCGCCCGT

CAC

>otu10

AAGTCGAACGGTGAACACGGAGCTTGCTCTGTGGGATCAGTGGCGAACGGGTGAGTAACA

CGTGAGCAACCTGCCCCTGACTCTGGGATAAGCGCTGGAAACGGCGTCTAATACTGGATA

TGTGACGTGACCGCATGGTCTGCGTTTGGAAAGATTTTTCGGTTGGGGATGGGCTCGCGG

CCTATCAGCTTGTTGGTGAGGTAATGGCTCACCAAGGCGTCGACGGGTAGCCGGCCTGAG

AGGGTGACCGGCCACACTGGGACTGAGACACGGCCCAGACTCCTACGGGAGGCAGCAGTG

GGGAATATTGCACAATGGGCGAAAGCCTGATGCAGCAACGCCGCGTGAGGGATGACGGCC

TTCGGGTTGTAAACCTCTTTTAGCAGGGAAGAAGCGAAAGTGACGGTACCTGCAGAAAAA

GCGCCGGCTAACTACGTGCCAGCAGCCGCGGTAATACGTAGGGCGCAAGCGTTATCCGGA

ATTATTGGGCGTAAAGAGCTCGTAGGCGGTTTGTCGCGTCTGCTGTGAAATCCCGAGGCT

CAACCTCGGGCCTGCAGTGGGTACGGGCAGACTAGAGTGCGGTAGGGGAGATTGGAATTC

CTGGTGTAGCGGTGGAATGCGCAGATATCAGGAGGAACACCGATGGCGAAGGCAGATCTC

TGGGCCGTAACTGACGCTGAGGAGCGAAAGGGTGGGGAGCAAACAGGCTTAGATACCCTG

GTAGTCCACCCCGTAAACGTTGGGAACTAGTTGTGGGGTCCATTCCACGGATTCCGTGAC

GCAGCTAACGCATTAAGTTCCCCGCCTGGGGAGTACGGCCGCAAGGCTAAAACTCAAAGG

AATTGACGGGGACCCGCACAAGCGGCGGAGCATGCGGATTAATTCGATGCAACGCGAAGA

ACCTTACCAAGGCTTGACATATACGAGAACGGGCCAGAAATGGTCAACTCTTTGGACACT

CGTAAACAGGTGGTGCATGGTTGTCGTCAGCTCGTGTCGTGAGATGTTGGGTTAAGTCCC

GCAACGAGCGCAACCCTCGTTCTATGTTGCCAGCACGTAATGGTGGGAACTCATGGGATA

CTGCCGGGGTCAACTCGGAGGAAGGTGGGGATGACGTCAAATCATCATGCCCCTTATGTC

TTGGGCTTCACGCATGCTACAATGGCCGGTACAAAGGGCTGCAATACCGTGAGGTGGAGC

GAATCCCAAAAAGCCGGTCCCAGTTCGGATTGAGGTCTGCAACTCGACCTCATGAAGTCG

GAGTCGCTAGTAATCGCAGATCAGCAACGCTGCGGTGAATACGTTCCCGGGTCTTGTACA

CAC

>otu11

AAGTCGAGCGGTAAGGCCCTTCGGGGTACACGAGCGGCGAACGGGTGAGTaACACGTGGG

TGATCTGCCTCGCACTTCGGGATAAGCCTGGGAAACTGGGTCTAATACCGGATACGACTC

CTGGTCGCATGACCGGGAGTGGAAAGATTTATCGGTGCGAGATGAGCCCGCGGCCTATCA

GCTTGTTGGTGGGGTAAAGGCCTACCAAGGCGACGACGGGTAGCCgGCCTGAGAGGGCGA

CCGGCCACACTGGGACTGAGACACGGCCCAGACTCCTACGGGAGGCAGCAGTGGGGAATA

TTGCACAATGGGCGAAAGCCTGATGCAGCGACGCCGCGTGAGGGATGACGGCCTTCGGGT

TGTAAACCTCTTTCGACTCCGACGAAGCGCAAGTGACGGTAGGAGTAGAAGAAGCACCGG

CCAACTACGTGCCAGCAGCCGCGGTAATACGTAGGGTGCGAGCGTTGtCCGGAATTACTG

GGCGtAAAGAGCTTGTAGGCGGTTtGTCGCgTCGTCTGTGAAAACTCACAGcTCAACTGT

GAGCTTGCAGGCGATAcGGGCAGACTTGAGTACTGCAGGGGAGACTGGAATTCCTGGTGT

AGCGGtGGAATGCGCAGATATCAGGAGGAACACCGGTGGCGAAGGCGGGTCTCTGGGCAG

TAACTGACGCTGAGAAGCGAAAGCGTGGGTAGCGAACaGGATTAGAtACCCTGGTAGTCC

ACGCCGTAAACGGTGGGCGCTAGGTGTGGGTTTCCTTCCACGGGATCCGTGCCGTAGCTA

ACGCATTAAGCGCCCCGCCTGGGGAGTACGgCCGCAAGGCTAAAACTCAAAGGAATTGAC

GGGGGCCCGCACAAGCGGCGGAGCATGTGGATTAATTCGATGCAACGCGAAGAACCTTAC

CTGGGTTTGACATACACCGGAAACCTGCAGAGATGTAGGCCCCCTTGTGGCCGGTGTACA

GGTGGTGCATGGCTGTCGTCAGCTCGTGTCGTGAGATGTTGGGTTAAGTCCCGCAACGAG

CGCAACCCTTGTCCTGTGTTGCCAGCGCGTAAAGGCGGGGACTCGCAGGAGACTGCCGGG

GTCAACTCGGAGGAAGGTGGGGACGACGTCAAGTCATCATGCCCCTTATGTCCAGGGCTT

CACACATGCTACAATGGCCGGTACAGAGGGCTGCGATACCGCAAGGTGGAGCGAATCCCT

TAAAGCCGGTCTCAGTTCGGATCGGGGTCTGCAACTCGACCCCGTGAAGTTGGAGTCGCT

AGTAATCGCAGATCAGCAACGCTGCGGTGAATaGGGTCCCGGGCCTTGTACACACCGCCC

GTC

>otu12

TGCAAGTCGAGCGGAAGGCCCTTCGGGGTACTCGAGCGGCGAACGGGTGAGTAACACGTG

AGCAACCTGCCCCAGGCTTTGGGATAACCCCGGGAAACCGGGGCTAATACCGGATATGAC

CTTGCACCGCATGGTGTTTGGTGGAAAGTTTTTCGGCTTGGGATGGGCTCGCGGCCTATC

AGCTTGTTGGTGGGGTGATGGCCTACCAAGGCGACGACGGGTAGCCGGCCTGAGAGGGCG

ACCGGCCACACTGGGACTGAGACACGGCCCAGACTCCTACGGGAGGCAGCAGTGGGGAAT

ATTGCACAATGGGCGGAAGCCTGATGCAGCGACGCCGCGTGAGGGATGACGGCCTTCGGG

TTGTAAACCTCTTTCAGCAGGGACGAAGCGTAAGTGACGGTACCTGCAGAAGAAGCGCCG

GCCAACTACGTGCCAGCAGCCGCGGTAAGACGTAGGGCGCGAGCGTTGTCCGGATTTATT

GGGCGTAAAGAGCTCGTAGGCGGCTTGTCGCGTCGACCGTGAAAACCTGGGGCTCAACCC

CAGGCCTGCGGTCGATACGGGCAGGCTAGAGTTCGGTAGGGGAGACTGGAATTCCTGGTG

TAGCGGTGAAATGCGCAGATATCAGGAGGAACACCGGTGGCGAAGGCGGGTCTCTGGGCC

GATACTGACGCTGAGGAGCGAAAGCGTGGGGAGCGAACAGGATTAGATACCCTGGTAGTC

CACGCTGTAAACGTTGGGCGCTAGGTGTGGGGGGCCTCTCCGGTTCCCTGTGCCGCAGCT

AACGCATTAAGCGCCCCGCCTGGGGAGTACGGCCGCAAGGCTAAAACTCAAAGGAATTGA

CGGGGGCCCGCACAAGCGGCGGAGCATGCGGATTAATTCGATGCAACGCGAAGAACCTTA

CCTGGGTTTGACATGGCCGCAAAACTGTCAGAGATGGCAGGTCCTTCGGGGGCGGTCACA

GGTGGTGCATGGCTGTCGTCAGCTCGTGTCGTGAGATGTTGGGTTAAGTCCCGCAACGAG

CGCAACCCTCGTTCGATGTTGCCAGCGCGTTATGGCGGGGACTCATCGAAGACTGCCGGG

GTCAACTCGGAGGAAGGTGGGGATGACGTCAAGTCATCATGCCCCTTATGTCCAGGGCTT

CACGCATGCTACAATGGCCGGTACAATGGGCTGCGATACCGTGAGGTGGAGCGAATCCCA

AAAAGCCGGTCTCAGTTCGGATCGGGGTCTGCAACTCGACCCCGTGAAGTCGGAGTCGCT

AGTAATCGCAGATCAGCAACGCTGCGGTGAATACGTTCCCGGGCCTTGTACACACCGCCC

GTC

>otu13

CCTTCGGtGtGGATTAGTGGCGAACGGGTGAGTaaCACGTGGGCAATCTGCCCTGCACTC

TGGGACAAGCCCTGGaAACGGGGTCTAATACCGGATACTGATCGCCTTGGGCATCCTTGG

TGATCGAAAGCTCCGGCGGTGCAGGATGAGCCCGCGGCCTATCAGCTTGTTGGTGAGGTA

ATGGCTCACCAAGGCGACGACGGGTAGCCGGCCTGAGAGGGCGACCGGCCACACTGGGAC

TGAGACACGGCCCAGACTCCTaCGGGAGGCAGCAGTGGGGAATATTGCACAATGGGCGAA

AGCCTGATGCAGCGACGCCGCGTGAGGGATGACGGCCTTCGGGTTGTAAACCTCTTTCAG

CAGGGAAGAAGCGAAAGTGACGGTACCTGCAGAAGAAGCGCCGGCTAACTACGTGCCAGC

AGCCGCGGTAATACGTAGGGCGCGAGCGTTGTCCGGAATTATTGGGCGTAAAGAGCTCGT

AGGCGGCTTGTCGCGTCGGTTGTGAAAGCCCGGGGCTTAACCCCGGGTCTGCAGTCGATA

CGGGCAGGCTAGAGTTCGGTAGGGGAGATCGGAATTCCTGGTGTAGCGGTGAAATGCGCA

GATATCAGGAGGAACACCGGTGGCGAAGGCGGATCTCTGGGCCGATACTGACGCTGAGGA

GCGAAAGCGTGGGGAGCGAACAGGATTAGATACCCTGGTAGTCCACGCCGTAAACGGTGG

GCACTAGGTGTGGGCGACATTCCACGTCGTCCGTGCCGCAGCTAACGCATTAAGTGCCCC

GCCTGGGGAGTACGGCCGCAAGGCTAAAACTCAAAGGAATTGACGGGGGCCCGCACAAGC

GGCGGAGCATGTGGCTTAATTCGACGCAACGCGAAGAACCTTACCAAGGCTTGACATACA

CCGGAAAACCCTGGAGACAGGGTCCCCCTTGTGGTCGGTGTACAGGTGGTGCATGGCTGT

CGTCAGCTCGTGTCGTGAGATGTTGGGTTAAGTCCCGCAACGAGCGCAACCCTTGTCCCG

TGTTGCCAGCAGGCCCTTGTGGTGCTGGGGACTCACGGGAGACCGCCGGGGTCAACTCGG

AGGAAGGTGGGGACGACGTCAAGTCATCATGCCCCTTATGTCTTGGGCTGCACACGTGCT

ACAATGGCCGGTACAATGAGCTGCGATACCGCGAGGTGGAGCGAATCTCAAAAAGCCGGT

CTCAGTTCGGATTGGGGTCTGCAACTCGACCCCATGAAGTCGGAGTCGCTAGTAATCGCA

GATCAGCATTGCTGCGGTGAATACGTTCCCGGGCCTTGTACACACCGCCCGTCACGTCAC

GAA

>otu14

ACATGCAGTCGAACGATGAACCTCCTTCGGGAGGGGATTAGTGGCGAACGGGTGAGTAAC

ACGTGGGCAATCTGCCCTGCACTCTGGGACAAGCCCTGGAAACGGGGTCTAATACCGGAT

ACGACCACTGAGGGCATCCTCGGTGGTGGAAAGCTCCGGCGGTGCAGGATGAGCCCGCGG

CCTATCAGCTTGTTGGTGGGGTGATGGCCTACCAAGGCGACGACGGGTAGCCGGCCTGAG

AGGGCGACCGGCCACACTGGGACTGAGACACGGCCCAGACTCCTACGGGAGGCAGCAGTG

GGGAATATTGCACAATGGGCGAAAGCCTGATGCAGCGACGCCGCGTGAGGGATGACGGCC

TTCGGGTTGTAAACCTCTTTCAGCAGGGAAGAAGCGAAAGTGACGGTACCTGCAGAAGAA

GCGCCGGCTAACTACGTGCCAGCAGCCGCGGTAATACGTAGGGCGCGAGCGTTGTCCGGA

ATTATTGGGCGTAAAGAGCTCGTAGGCGGCTTGTCACGTCGGTTGTGAAAGCCCGGGGCT

TAACCCCGGGTCTGCAGTCGATACGGGCAGGCTAGAGTTCGGTAGGGGAGATCGGAATTC

CTGGTGTAGCGGTGAAATGCGCAGATATCAGGAGGAACACCGGTGGCGAAGGCGGATCTC

TGGGCCGATACTGACGCTGAGGAGCGAAAGCGTGGGGAGCGAACAGGATTAGATACCCTG

GTAGTCCACGCCGTAAACGTTGGGCACTAGGTGTGGGCGACATTCCACGTCGTCCGTGCC

GCAGCTAACGCATTAAGTGCCCCGCCTGGGGAGTACGGCCGCAAGGCTAAAACTCAAAGG

AATTGACGGGGGCCCGCACAAGCGGCGGAGCATGTGGCTTAATTCGACGCAACGCGAAGA

ACCTTACCAAGGCTTGACATACACCGGAAAGCATCAGAGATGGTGCCCCCCTTGTGGTCG

GTGTACAGGTGGTGCATGGCTGTCGTCAGCTCGTGTCGTGAGATGTTGGGTTAAGTCCCG

CAACGAGCGCAACCCCTGTCCTGTGTTGCCAGCGGATCATGCCGGGGACTCACAGGAGAC

CGCCGGGGTCAACTCGGAGGAAGGTGGGGACGACGTCAAGTCATCATGCCCCTTATGTCT

TGGGCTGCACACGTGCTACAATGGCCGGTACAATGAGCTGCGATACCGCGAGGTGGAGCG

AATCTCAAAAAGCCGGTCTCAGTTCGGATTGGGGTCTGCAACTCGACCCCATGAAGTCGG

AGTCGCTAGTAATCGCAGATCAGCATTGCTGCGGTGAATACGTTCCCGGGCCTTGTACAC

ACC

>otu15

ACATGCAGTCGAACGATGATCTCCCGCTTGCGGGGGTGATTAGTGGCGAACGGGTGAGTA

ATACGTGAGTAACCTGCCCTTGACTCTGGGATAAGCCTGGGAAACCGGGTCTAATACTGG

ATACTACTTCCTGCCGCATGGTGGGGGGTGGAAAGGGTTcTACTGGTTTTGGATGGGCTC

ACGGCCTATCAGCTTGTTGGTGGGGTAATGGCTCACCAAGGCGACGACGGGTAGCCGGCC

TGAGAGGGTGACCGGCCACACTGGGACTGAGACACGGCCCAGACTCCTACGGGAGGCAGC

AGTGGGGAATATTGCACAATGGGCGGAAGCCTGATGCAGCGACGCCGCGTGAGGGATGAC

GGCCTTCGGGTTGTAAACCTCTTTCAGCAGGGAAGAAGCCACAAGTGACGGTACCTGCAG

AAGAAGCGCCGGCTAACTACGTGCCAGCAGCCGCGGTAATACGTAGGGCGCAAGCGTTGT

CCGGAATTATTGGGCGTAAAGAGCTCGTAgGcGGTTTGTCGCGTCTGCTGTGAAAGCCCG

GGGCTCAACCCCGGGTCTGCAGTGGGTACGGGCAGACTAGAGTGCAGTAGGGGAGACTGG

AATTCCTGGTGTAGCGGTGAAATGCGCAGATATCAGGAGGAACACCGATGGCGAAGGCAG
GTCTCTGGGCTGTTACTGACGCTGAGGAGCGAAAGCATGGGGAGCGAACAGGATTAGATA
CCCTGGTAGTCCATGCCGTAAACGTTGGGCACTAGGTGTGGGGGACATTCCACGTTCTCC
GCGCCGTAGCTAACGCATTAAGTGCCCCGCCTGGGGAGTACGGCCGCAAGGCTAAAACTC
AAAGGAATTGACGGGGGCCCGCACAAGCGGCGGAGCATGCGGATTAATTCGATGCAACGC
GAAGAACCTTACCAAGGCTTGACATTCACCGGACCGCACTGGAGaCAGTGCTTCCCTTCG
GGGTCGGTGGACAGGTGGTGCATGGTTGTCGTCAGCTCGTGTCGTGAGATGTTGGGTTAA
GTCCCGCAACGAGCGCAACCCTCGTTCTATGTTGCCAGCACGTGATGGTGGGGACTCATA
GGAGACTGCCGGGGTCAACTCGGAGGAAGGTGGGGATGACGTCAAATCATCATGCCCCTT
ATGTCTTGGGCTTCACGCATGCTACAATGGCCGGTACAAAGGGTTGCGATACTGTGAGGT
GGAGCTAATCCCAAAAAGCCGGTCTCAGTTCGGATTGAGGTCTGCAACTCGACCTCATGA
AGTCGGAGTCGCTAGTAATCGCAGATCAGCAACGCTGCGGTGAATACGTTCCCGGGCCTT
GTA
